# OSAnalyzer: A Bioinformatics Tool for the Analysis of Gene Polymorphisms Enriched with Clinical Outcomes

**DOI:** 10.3390/microarrays5040024

**Published:** 2016-09-23

**Authors:** Giuseppe Agapito, Cirino Botta, Pietro Hiram Guzzi, Mariamena Arbitrio, Maria Teresa Di Martino, Pierfrancesco Tassone, Pierosandro Tagliaferri, Mario Cannataro

**Affiliations:** 1 Department of Medical and Surgical Science, University Magna Graecia of Catanzaro, Catanzaro 88100, Italy; hguzzi@unicz.it (P.H.G.); cannataro@unicz.it (M.C.); 2 Department of Experimental Medicine and Clinic, University Magna Graecia of Catanzaro, Catanzaro 88100, Italy; cirino.botta@gmail.com (C.B.); teresadm@unicz.it (M.T.D.M.); tassone@unicz.it (P.Tas.); tagliaferri@unicz.it (P.Tag.); 3 ISN-CNR, Roccelletta di Borgia, Catanzaro 88100, Italy; mariamena.arbitrio@cnr.it

**Keywords:** genotyping microarrays, ADME genes, pharmacogenomics, overall survival, progression-free survival

## Abstract

Background: The identification of biomarkers for the estimation of cancer patients’ survival is a crucial problem in modern oncology. Recently, the Affymetrix DMET (Drug Metabolizing Enzymes and Transporters) microarray platform has offered the possibility to determine the ADME (absorption, distribution, metabolism, and excretion) gene variants of a patient and to correlate them with drug-dependent adverse events. Therefore, the analysis of survival distribution of patients starting from their profile obtained using DMET data may reveal important information to clinicians about possible correlations among drug response, survival rate, and gene variants. Methods: In order to provide support to this analysis we developed OSAnalyzer, a software tool able to compute the overall survival (OS) and progression-free survival (PFS) of cancer patients and evaluate their association with ADME gene variants. Results: The tool is able to perform an automatic analysis of DMET data enriched with survival events. Moreover, results are ranked according to statistical significance obtained by comparing the area under the curves that is computed by using the log-rank test, allowing a quick and easy analysis and visualization of high-throughput data. Conclusions: Finally, we present a case study to highlight the usefulness of OSAnalyzer when analyzing a large cohort of patients.

## 1. Introduction

The possibility to integrate clinical data with high-throughput data at the single patient level has gained increasing interest in different fields of medicine [1]. Indeed, clinicians can use this new integrated data to evaluate, in a more comprehensive way, the efficacy of the therapy in a cohort of patients, to draw more detailed conclusion on the benefits of the treatment, or to modify the drug dosage to reduce the side effects by following the genomic features of each patient and improving the efficacy of the treatment [2]. This scenario, however, introduces new challenges from a computational point of view, since the high-throughput technologies such as Next Generation Sequencing (NGS) and Genome Wide-Association Studies (GWAS), are changing clinical medicine in a data-driven science [3]. This event requires the development of new, efficient algorithms and software platforms to analyze this enormous amount of data in the shortest time possible [4,5,6,7,8]. Consequently, researchers can obtain a broader and more detailed perspective of the study under investigation, allowing them to link together genomic variants with clinical outcomes, including the response to certain treatments and prognosis of the patients under specific clinical studies [9,10,11]. Researchers focus on the occurrence/observation of an event of interest, aiming to develop predictive models. The outcome for a patient is the occurrence/observation of an event of interest. For example, the event may be: (i) the metastatic spread of a particular type of cancer; (ii) death, from any cause, of patients in a trial study of different treatments for lung cancer; and (iii) death due to lung cancer during the trial study. The events in these examples present differences in terms of certainty that can be attributed to their observation. In (i) it could be very difficult to be certain; in (ii) it is unequivocal; whereas in (iii) it might be surprisingly uncertain. In each of these examples, the patients are usually observed over a specified period of time, focusing on the time at which the event of interest occurs. Time to event is a clinical course duration variable for each patient with a beginning and an end, known as the survival time, or even failure time. For example, it may begin when the subject is enrolled into a study or when the event of interest is reached, or the patient is dropped out of the study for some unknown reasons, known as censored. All incomplete survival information are considered censored, including patients that, at the moment of the analysis, have still not reached the event. Censoring is an important issue in survival analysis, representing a particular type of missing data [12], needing to be handled in a proper way. Survival analysis is composed of a set of statistical methods able to estimate lifetime between two clearly defined events known as time of response or time of failure. The more popular survival analysis that estimates the probability of survival is the Kaplan-Meier method (K and M) [13], also called the product limit estimator. The Kaplan-Meier method allows to easily handling censored data. The K and M estimator is used to obtain univariate descriptive statistics for survival data, including the median survival time, and to compare the survival experience for two or more groups. To compare the overall differences between estimated survival curves of two or more groups of subjects, such as males versus females, or treated versus untreated (control) groups, several tests are available, including the log-rank test [14]. The comparison of survival curves makes it possible to reveal if the treatment was effective in terms of increasing the overall survival of a group of subjects or if the differences observed were simply the result of chance, for example.

In particular, in this work we focus on the comparison of survival analysis of patients and on the correlation of these analysis to the genomic characteristics of patients analyzed by DMET microarray technology. We provide an automatic analysis methodology to compute the overall survival analysis (OS) and the progression-free survival (PFS) from a whole DMET dataset produced by using the Affymetrix DMET PLUS platform and successively extended by adding temporal data.

The main aim of OSAnalyzer is to automatize and simplify the work of biomedical researchers and clinical researchers when interpreting and analyzing the data of observational studies.

The rest of the paper is structured as follows: Section 2 introduces the data provided by the DMET platform and the OS-dataset obtained by merging DMET data with clinical data, Section 2.2 investigates the current state of the art in analyzing the correlation among survival data and genomic features, Section 3 presents the OSAnalyzer tool and describes its use through a case study by using the OSAnalyzer tool, Section 4 discusses the main results of the proposed methodology of automatic analysis of genomics data integrated with clinical data and, finally, Section 5 concludes the paper and outlines future works.

## 2. Materials and Methods

OSAnalyzer is a software tool for the automatic association analysis among the genome variants of patients and their clinical condition, e.g., the different responses to drugs. OSAnalyzer allows for automatically computing the OS and PFS analysis of a whole DMET dataset previously annotated by adding the clinical data of patients.

OSAnalyzer is based upon suitable data structures and algorithms able to optimize the computational effort necessary to compute the OS and PFS of a whole DMET dataset encompassing temporal events (such events are usually added to the DMET data only after the DMET analysis has been performed).

Files produced by using the DMET platform are in a binary microarray data format (e.g., a Affymetrix Probe Results File in Computing category (CEL) Affymetrix raw data file for each sample) to obtain a single table of alleles must be used by the DMET console tool to aggregate data coming from all samples of a dataset. Moreover, the DMET console can export an SNP table in tabular-delimited text file format (.txt), a standard format for text files, or exported as standard Excel files (.xls). In detail, the first row contains the identifiers of the samples while the first column contains the identifiers of the probes. DMET console-exported outcomes are arranged as a large n × m matrix of single nucleotide polymorphisms (SNPs), where *n* is the number of probes (*n =* 1936 for the current DMET chips) and *m* is the number of samples (patients), as depicted in Table 1.

A generic element (*i*,*j*) in Table 1 contains the *i*-th identified SNP in the *j*-th sample, so it has the form *X*/*Y*, where *X*, *Y* ∈ {A,T,C,G,-}.

In order to make the automatic computation of OS and PFS possible for OSAnalyzer, it was necessary to extend the output produced by the DMET platform by adding clinical information after the header row. For each sample (patient), the supplementary information are, temporal information i.e., the period between the start and end of the clinical observation along with a status, indicating whether or not each patient had a clinical event of interest, e.g., death or metastasis. The extended dataset, hereinafter called OS-dataset, is arranged as conveyed in Table 2.

After annotating a DMET dataset, all the information necessary to compute the OS and PFS curves are available. The curves are obtained by implementing the well-known methodology defined by Kaplan and Meier [13]. The K and M estimator is a non-parametric statistic used to estimate the survival function *S(t)*, which is defined as the probability that an individual survives more than time *t*.

The survival function *S(t)* is computed for each time interval, in particular, survival probability is calculated as the ratio between the number of surviving patients and the number of at-risk patients. Patients who have died, dropped out, or that did not reach the time yet, are not counted as at-risk. Patients who are lost for some reason are considered censored and are not included in the denominator. The probability of surviving to any point is estimated from the cumulative probability of surviving each of the preceding time intervals (calculated as the product of preceding probabilities). The probability of surviving from *0* to *t*_1_ is then estimated by Equation (1):

(1) $$S\left(t_{1}\right)=1\;-\;\frac{d_{1}}{r_{1}}$$

where the ratio $\frac{d_{1}}{r_{1}}$ is the estimated proportion of dying in that interval. The estimated survivor function for any arbitrary time *t* is given by Equation (2):

(2) $$S\left(t\right)=\prod_{1}^{N}1-\frac{d_{i}}{r_{i}}$$

It is worth noting that we are considering the product of all *i* values for which *t*’ is less than the time *t* (which is the number of patient at risk at time *t*’).

### 2.1. OSAnalyzer and SNPs Handling for Computing Kaplan-Meier

**Figure 1:**
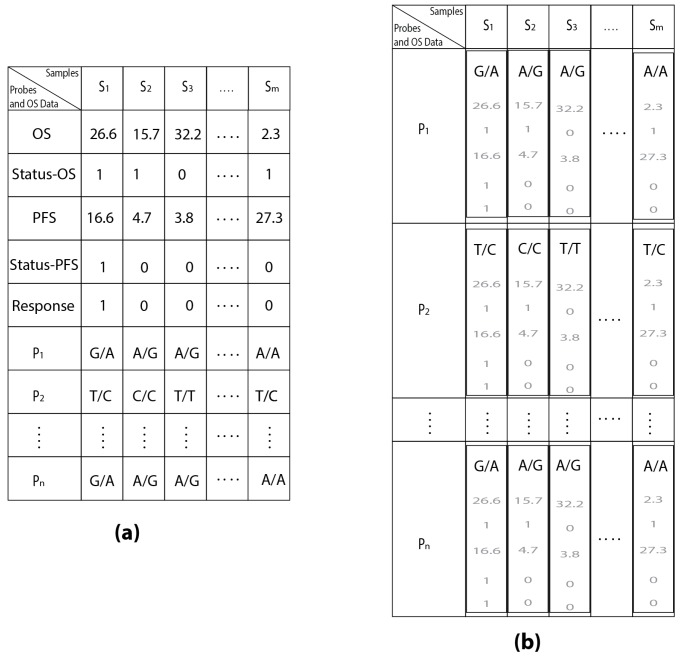
The use of VPF applied to the OS-dataset (**a**); the produced result is shown in (**b**); the survival data are faded because they are only linked and not replicated for each SNP into the table.

The identifiers of SNPs (i.e., A/G, G/A, and so on) typical for the OS-dataset represented in Table 2 are used to divide a generic probe into groups of samples that have the same SNP, but are not useful to estimate K and M. At most three groups of samples can be presented in each probe, given that the possible combinations of SNPs for each probe are equal to three (i.e., if the probe contains the alleles A and G then the possible combinations will be A/A, G/A, and G/G). In this way, for each probe it will be possible to group together the samples with the same polymorphism and evaluate the survival of the samples belonging to the identified groups. Before creating groups of samples identified by the same SNP, it is essential to link together all the SNPs and survival data for the same sample. This connection was realized by defining a virtual projection function (VPF) able to link together the survival data of each subject with their SNPs. The VPF virtually links together the survival data with all the SNPs belonging to the same sample without replicating the overall survival data for all the 1936 SNP for each subject, as conveyed in Figure 1.

At the same time this tight coupling between SNPs and the related survival data make it possible to split each probe into groups and accurately compute the K and M estimator for each identified group. By means of the VPF, OSAnalyzer is able to compute K and M by using only temporal data without needing to convert the literal SNPs to numerical values.

### 2.2. Related Works

In this section we highlight the main features and capabilities of several available software tools to analyze and validate survival data.

Below, the main features and capabilities of some well-known stand-alone and web-based tools used to analyze survival data are summarized.

Partial Cox regression analysis (PCR) [15] is a collection of command line R scripts written using the R code language. Partial Cox regression analysis is based on a partial Cox regression method for constructing mutually uncorrelated components based on microarray gene expression data for predicting the survival of future patients. The R code of PCR is available upon request.

Significance analysis of microarrays (SAM) [16] is a statistical software for finding significant genes in a set of microarray experiments. The data should be put in an Excel spreadsheet and arranged as follows: the first row of the spreadsheet has information about the response variable, whereas all remaining rows contain gene expression data, one row per gene. The response variable may be a grouping like (untreated, treated), a quantitative variable (like blood pressure), or a possibly censored survival time. SAM is used to identify genomic features correlated with biological and/or clinical phenotypes of interest, including time-to-event clinical outcome. SAM is freely available after registration as an R package or Excel add-in.

GenePattern [17] is a software based on a simple graphical user interface (GUI) used to provide access to a broad number of computational methods represented to the user as graphical modules, used to analyze genomic data. To analyze a particular dataset, users have to manually define a customized pipeline using the available modules. Finally, among the other statistical capabilities provided by GenePattern, it is possible to create and visualize survival curves based on censored data arranged in a “cls” file. GenePattern is freely available, but before the first run the GenePattern registration page will appear in your browser (modules run only on the GenePattern server).

PSPP (https://www.gnu.org/software/pspp/) is a free software application (free alternative to SPSS) for statistical analysis of sampled data. It comes with a graphical user interface making it easy to use the available scientific capabilities. Furthermore, for the advancer user, it is possible to use PSPP by the command line interface to obtain better performance.

R (https://www.r-project.org) is an open source software environment for statistical and mathematical computing and graphics. Moreover, R can be considered as a different implementation of the S language, making it possible to write code that runs under R.

SPSS (http://www-01.ibm.com/software/analytics/spss/) is a software package developed to help users in statistical data analysis process. In addition to statistical analysis SPSS provide to the users data mining, text analytics, and data collection capabilities.

MATLAB (www.mathworks.com/products/matlab/) (matrix laboratory) is a numerical computing environment. MATLAB allows a variety of statistical tool to manipulate data and graphical techniques to produce quality plots. On the other hand, MATLAB introduces its proprietary programming language with which it is possible to use all the functionalities available to MATLAB, including the implementation of algorithms, creation of user interfaces, and so on.

Kaplan-Meier Plotter [18] is an integrated database (containing breast, ovarian, and lung cancer information) and an online tool capable of uni/multivariate analysis for in silico validation of new biomarker candidates in non-small cell lung cancer. Univariate and multivariate Cox regression analysis, Kaplan-Meier survival plots with hazard ratio and log-rank *p*-values are calculated and plotted by using R.

Net-Cox [19] is a network-based Cox regression model used for a large-scale survival analysis across multiple ovarian cancer datasets. Datasets have to be arranged as a tabular delimited text file in order to be compatible with Net-Cox. In particular, compatible datasets are obtained by combining cancer information and patient clinical information stored in separate files into a unique file arranged as textual matrix. Net-Cox is freely available as a MATLAB plug-in.

Survcomp [20] is an R-based Bioconductor freely-available package for survival risk model comparison. The survcomp package provides functions to assess and compare the performance of risk prediction (survival) models.

cBioPortal [21] is a web-based resource for cancer genomics providing visualization, analysis, and downloading of large-scale cancer genomics datasets. All functionalities in cBioPortal visualization, querying, and analysis in cBioPortal are easy to use by means of a graphical user interface, including the analysis of survival data analysis based on Kaplan-Meier, and log-rank test to compare multiple survival curves. cBioPortal is also available as CGDS-R/MATLAB packages. Providing a basic set of functions for querying the cancer genomic data server (CGDS) via the R/MATLAB platform for statistical computing.

PrognoScan [10] is a freely-available web-database usable through a simple graphical user interface. PrognoScan makes it easy for users to search the relation between gene expression and patient prognosis, such as overall survival (OS) and disease-free survival (DFS) across a large collection of publicly available cancer microarray datasets. PrognoScan includes the Kaplan-Meier estimator and the plotter function to plot Kaplan-Meier curves.

SurvExpress [22] is a comprehensive gene expression database and web-based tool providing survival analysis and risk assessment in cancer datasets using a biomarker gene list as: Kaplan-Meier plots, the log-rank test of differences between groups, and the hazard ratio estimate.

All of the tools listed above have been developed for the analysis and visualization of gene expression data, with the exception of R, MATLAB, SPSS, and PSPP, which can be classified as general numerical computing environments.

Each tool used for analyzing input datasets requires that datasets meet specific criteria; otherwise the tools cannot analyze the data. For example, to analyze gene expression data with SAM data must be put into an Excel spreadsheet, where the first row contains the response measurements, one per column, starting from the third column. The remaining rows contain gene expression measurements, one row for every gene. “Column1” contains the gene name, “Column2” contains the Gene ID, whereas the remaining columns contain the expression measurements as numbers. Missing expression measurements should be reported as either blank or non-numeric values. It is worthy to note that the file format defined by the SAM tool is not compatible with the file formats defined by other software instruments. The tools developed to analyze gene expression data cannot deal with non-numerical value, limitation that highlights the impossibility for SAM and the other gene expression tools to analyze the DMET dataset since the DMET dataset contains only non-numerical values.

Different assessments must be made for general numerical analysis environments when they are used to analyze DMET datasets. Although they are numerical analysis tools they are not intended to work directly with SNP datasets, the reason for which SPSS, R, and so forth can analyze OS-datasets only after the dataset has been manually converted in a format compatible with the tool used for the analysis. To the best of our knowledge, there are currently no tools for import/export of DMET/OS dataset in R, SPSS, MATLAB, and so on. For example, analyzing an OS-dataset with SPSS requires a significant effort for the users because he/she has to manually convert each of 1936 probes (rows) times the number of subjects (columns). Whereby, an OS-dataset with 100 subjects requires 193,600 conversions to convert each SNP into a numerical value (i.e., assigning the value 1 to A/A, 2 to A/C and so on). Moreover, in addition to translation of SNP, users must take into account the removal of special symbols such as “NoCall”, “ZeroCopyNumber”, “RareAllele”, and so on, because they are not useful for the data analysis. After this conversion step the user can upload the file in SPSS and must plot each probe individually, one after the other, in order to identify significant correlations between SNPs and overall survival. Another limitation of converting SNPs into numbers is that when users plot the overall survival curves, users lose the information about the SNP and the related curve. This correlation has to be remembered by the user that has to manually associate the numerical value to the plotted SNPs. Another way to analyze DMET data with general numerical analysis environments is to write a custom code able to import and convert the DMET dataset in a compatible format with the tool. This requires advanced programming skills and,it is time consuming and error prone. However this is a task not easy to pursue by life scientists, who only need to analyze data and to obtain clues about correlations among overall survival and SNP.

Conversely, OSAnalyzer is different from the tools listed above due to the fact that to the best of our knowledge, it is the unique that come with the capability to automatically analyze a whole DMET dataset annotated with clinical data. Automatic data analysis makes it possible to highlight which genomic features are useful for the association with clinical outcomes, including, for example, the response to certain treatments and prognosis of the patients under specific clinical scenarios. Thus, data analysis become straightforward, without users having to worry about how data should be arranged, which settings use or, even worse, manually investigate all the dataset to detect significant clues. Manual analysis is time-consuming and may increase the probability to introduce mistakes reducing the accuracy of the results. Instead, the use of OSAnalyzer avoids wasting time on the manual analysis of all probes in order to figure out which probes are relevant from an overall survival or PFS point of view [9,23]. On the other hand, the current version of OSAnalyzer cannot analyze gene expression data in order to plot overall survival curves and it presents limited data analysis functionalities.

**Figure 2:**
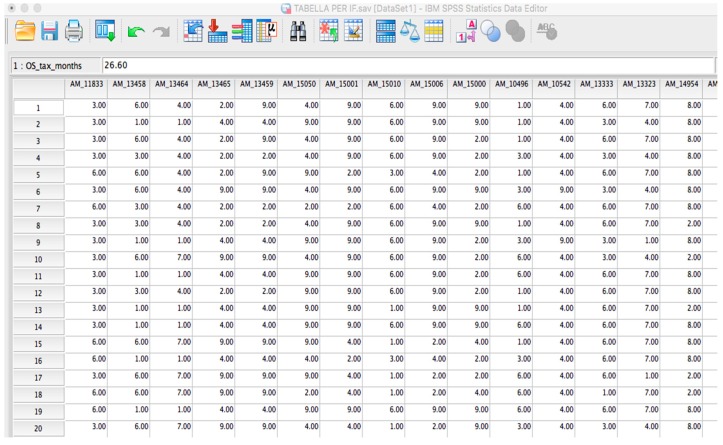
DMET dataset containing numerical SNPs and not literal SNPs.

Due to space limitation, we present only the comparison between OSAnalyzer and SPSS to prove the reliability of our tool. To be able to analyze OS-datasets with SPSS the first operation is to convert the literal SNP symbols contained into the OS-dataset into numerical values. To speed up the translation process, we used regular expressions to convert each SNP in the dataset into a unique numerical identifier. The translated file is depicted in Figure 2.

**Figure 3:**
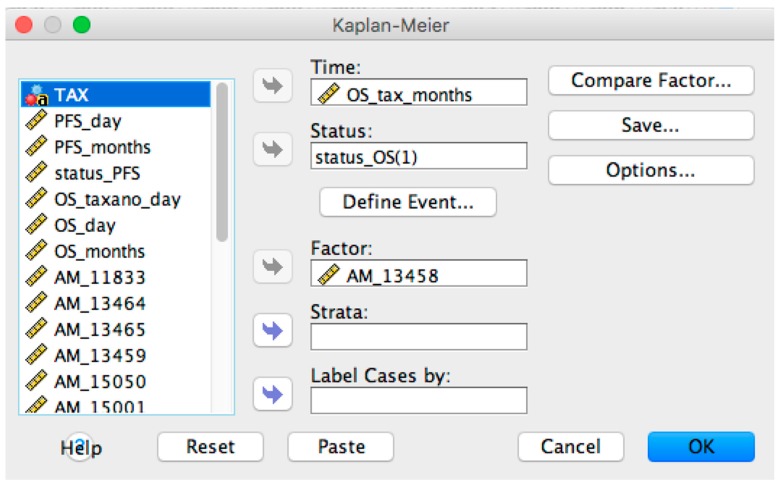
SPSS Kaplan-Meier configuration panel.

After loading the converted OS-dataset, the survival analysis in SPSS can be done by using the configuration panel, accessible from the menu “Analyze > Survival > Kaplan-Meier…” (see Figure 3).

**Figure 4:**
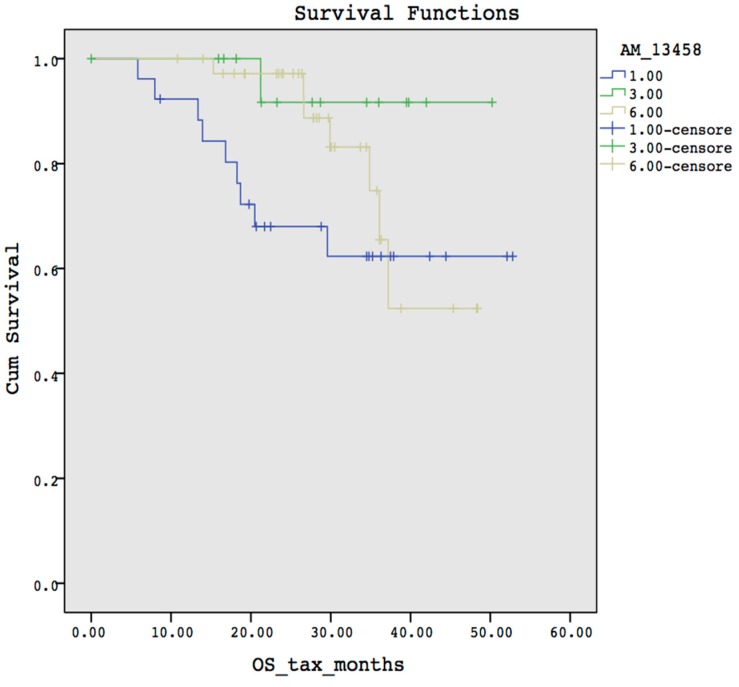
Survival curve obtained by using SPSS.

Subsequent to the selection of the Kaplan-Meier function, the user has to set up all of the configuration parameters and, in particular, has to enable the display of the survival curve from the “Options” menu. To finish the setup, the user has to click on the “OK” button to start the overall survival curve computation related to the selected probe (i.e., in this example the probe is AM_13458) and will be conveyed to the user as depicted in Figure 4. It is worthy to note that users have to manually investigate each probe one by one to identify significant correlation between SNPs and overall survival.

Conversely, using OSAnalyzer the same overall survival analysis requires less effort for the user. In fact, users have to load the whole OS-dataset and the tool conveys to the user the probe ranked accordingly to its statistical significance of the log-rank test. Finally, comparing the survival curves obtained by OSAnalyzer with the survival curves produced by SPSS can prove the reliability of OSAnalyzer.

**Figure 5:**
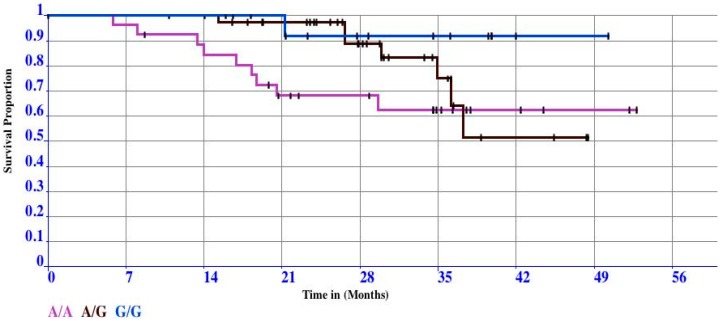
Survival curve obtained by using OSAnalyzer.

Both curves present the same trends, both have median values equal to 0, and censored data show the same distribution on each curve. Finally, both diagrams present a statistical significance equals to 0.087 meaning that the observed differences among the groups are due to the chance. Figure 4, obtained by using SPSS, is more complex to understand because it does not provide any information about which are the SNPs that belong to the probe AM_13458 due to the conversion step. In Figure 4 each curve is identified by means of the value “1.00, 3.00, 6.00”. Instead, in Figure 5, each curve is identified by the corresponding SNP, making it easier to identify which SNP is responsible for shorter survival, or toxicity, and so forth.

## 3. Results

This section describes the main features of the OSAnalyzer tool and presents an experimental case study of a genomics dataset annotated with clinical data by using OSAnalyzer.

### 3.1. OSAnalyzer

OSAnalyzer is a platform-independent application and it is entirely implemented using the Java 6 programming language, making it available for Windows, Linux, and MacOSX operating systems.

**Figure 6:**
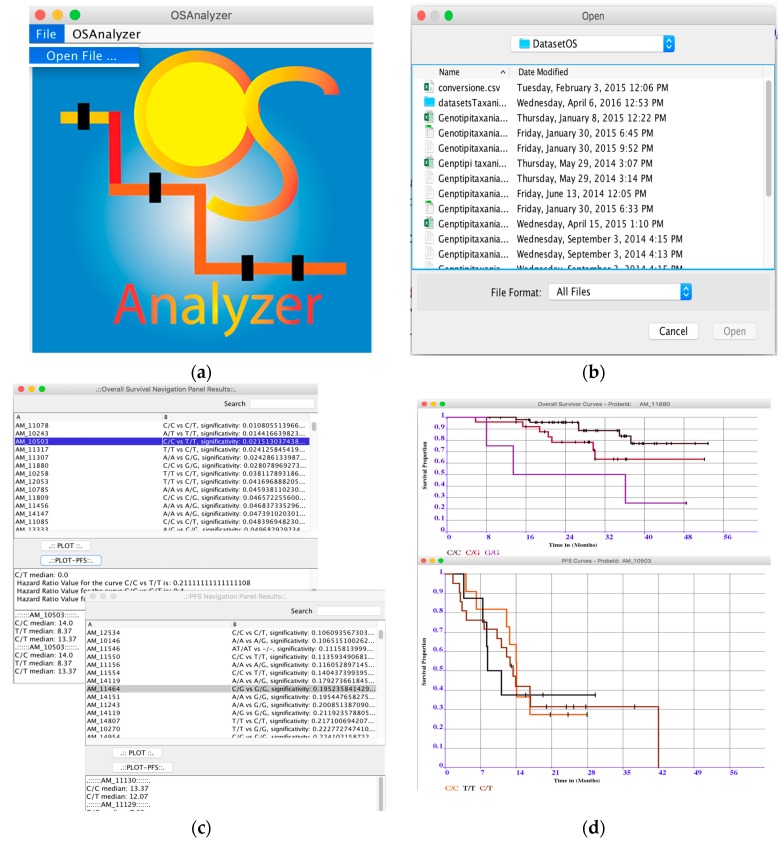
OSAnalyzer GUI. (**a**) OS-dataset loading menu; (**b**) OSAnalyzer file system navigation; (**c**) OS and PFS Navigation Panel Results; and (**d**) OS and PFS curves visualizer.

OSAnalyzer provides a simple and essential graphical user interface (GUI) allowing the users easy access to the tool`s functionalities. OSAnalyzer is very simple to use, the analysis of a complete OS-dataset requires just some clicks with the mouse, which are: (i) load the input OS-dataset by using the command “File” located in the menu bar as shown in Figure 6a; as result, OSAnalyzer shows to the user a file system navigation windows; (ii) browse the file system, in order to select the file to analyze (see Figure 6b); and (iii) wait as the OSAnalyzer finishes the computation and shows the results sorted in descending order according to the statistical significance of the log-rank test, and conveyed in two separate navigation panel results, one for OS, and one for PFS.

The use of two different navigation panel results makes it simple to locate the most relevant results for OS and PFS. Thus, users can easily find out the significance of a probe with respect to the OS and PFS (see Figure 6c). The main goal of the navigation panel results is to simplify the analysis of the results. In fact, each meaningful probe is related with three curves obtained by comparing the detected alleles in pairs, which present different values of significance. Thus, using the search function (locate in the top right corner of the window), it is possible to see the value of each curve (log-rank test) obtained comparing the three groups among them, Figure 6c. Selecting one probe per time and using the buttons “plot-os” or “plot-pfs” it is possible to see OS or PFS curves as depicted in Figure 6d. Moreover, at the bottom of the window, OSAnalyzer provides a quick summary of the most important measures for survival curves comparison such medians and the hazard ratio for each curve, as shown in see Figure 6d. The user can save the results and the curves that he/she considers relevant on file, by clicking with the right button of the mouse on the chart. Finally, OSAnalyzer can automatically annotate the relevant SNPs related to the overall survival by using annotation libraries provided by Affimetrix, or retrieving further information from dbSNP and PharmaGKB databases. OS-Analyzer is distributed under Creative Commons License, is freely downloadable for academic and not-for-profit institutions at: https://sites.google.com/site/overallsurvivalanalyzer/.

The automatic analysis of the whole microarray dataset avoids wasting time on the manual analysis of all probes in order to figure out which probes are relevant from an overall survival or PFS point of view. Users who are exploiting this feature of OSAnalyzer can automatically analyze a whole DMET microarray dataset in one go without further effort, as opposed to other available tools where the user is forced to manually organize the analysis of the whole dataset each time, increasing the possibility of introducing mistakes. This way, OSAnalyzer allows the users to focus only on the analysis of the results.

The capabilities made available by OSAnalyzer are:

- Loading and Analysis of OS-datasets: OSAnalyzer is currently able to parse information encoded in xlsx format (file format defined by Excel) and CSV (comma-separated value) data files, as well as tab-delimited files. This way users may also prepare their own dataset, e.g., merging together samples coming from different experimental batches;
- Overall survival significance: OSAnalyzer automatically computes and visualizes the overall survival significance related with all probes, showing to the users the probes ranked by *p*-value significance;
- Progression-Free Survival significance: OSAnalyzer automatically computes and visualizes the progression-free survival significance related with all probes, showing to the users the probes ranked by *p*-value significance;
- Overall and Progression-Free survival curves visualizer: it is possible to display the survival curve related with a selected probe. Furthermore, the current version of OSAnalyzer provides the users with additional information for the median related with each curve, the log-rank *p*-value and the hazard-ratio value;

### 3.2. Case Study

In this section, we will discuss K and M assessment in the context of overall survival before the event of interest, and how to read the results obtained from overall survival through a case study.

Generally, the purpose of the overall survival analysis is to employ the data available to provide assessment of the change of surviving to different times.

Clinical annotations are provided by the physicians and include overall survival, PFS, and response for each patient. These data are added to the DMET dataset (let see Table 1) to obtain the OS-dataset to correlate each SNP of the ADME genes to OS and PFS.

The OS-dataset (shown in Table 2) is achieved by adding clinical annotation (temporal clinical trends for each patient) that are: overall survival data (expressed in months) are collected from the starting point; for example, when the treatment starts or when the subject is enrolled into the study, to the end point that is, when the event of interest is reached i.e., dead. The occurrence of the event of interest is handled by using the Status-OS variable where 1 means the occurrence of the event of interest. Instead, 0 indicates censored data i.e., the subject is dropped out to the study for an unknown reason. PFS data (expressed in months) are collected for each subject, beginning when the subject starts the treatment and ending when the disease progresses or when the subject dies for any reason. PFS-Status variable takes a value of 1 that indicates the occurrence of the event of interest, whereas a value of 0 indicates a censored data. Finally, the response variable conveys the presence of metastasis when it assumes value equals to 1 and the absence of metastasis when it assumes value equals to 0.

In this way, OSAnalyzer can compute OS and PFS due to the presence/absence of SNP of the ADME genes for each probe, and, by using the log-rank test, it can compare and rank each SNP according to the *p*-value significance.

These results may help clinicians to understand if those SNPs may play a role in improving the response to cancer treatments and finally patients’ outcome.

A K and M estimator provides a graph of the survival function that summarizes the time-related information. To illustrate the OS analyses by using OSAnalyzer we generated a synthetic dataset (which is randomly generated) of 80 patients affected by an advanced cancer as the basis of an observational study of this disease.

Thus, survival analysis uses information from the whole follow-up period allowing us to illustrate the important point that comparative analysis between OS-curves depends upon the area under the K and M curves (AUC) and not only on differences based on single points, especially in real clinical studies.

The first step of the K and M analysis concerns with the data collection and arrangement. Data arrangement is necessary to make data in an appropriate format expected from the chosen analysis tool. There exist plenty of statistical analysis tools, available under GNU General Public License such as OSAnalyzer (https://sites.google.com/site/overallsurvivalanalyzer/), PSPP, or proprietary software, such as SPSS and MATLAB, each one with its requirements in terms of data arrangement. All software cited above require that data be arranged in a tabular form, containing, at least, the following information: (i) serial times; (ii) status at serial time (1 event of interest; 0 censored); and (iii) other kinds of clinical data, such as response rate, istotype, sex, etc., as nominal variables.

In any case, before beginning the analysis of the data, it is necessary to choose the analysis tool. The choice must be made according to the type of data to analyze. For instance, to analyze an SNP DMET dataset with SPSS, the user is required an extra effort to convert each single SNP “A/A, A/T, ...” in numerical values, given the impossibility of SPSS to analyze string values. Such conversion must be done manually by the user, increasing the probability of introducing errors due to the manual translation. It is worthy to note here that the translated file is necessary even for the PSPP software tool. To avoid this expensive step, OSAnalyzer can automatically analyze such SNP DMET datasets, and, most importantly, provide to the users all of the results ranked accordingly to the statistical significance of log-rank test.

**Figure 7:**
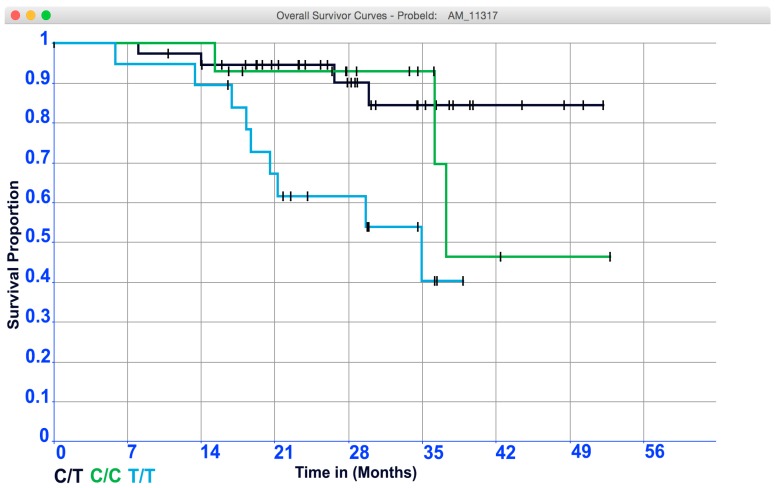
The K and M curve obtained analyzing the OS-dataset under investigation.

To illustrate how this all works, we prepared a synthetic OS-dataset extended with temporal data related to the subjects in each of the three groups related to the 3 allele variants in each probe (total of 80 subjects). The event of interest is “death” represented by the symbol 1. To understand the K and M curves let us look to Figure 7.

The lengths of the horizontal lines along the *X*-axis represent the duration of the survival time. The slopes indicate the end of an interval, due to the occurrence of the event of interest. The vertical lines have an aesthetic function only because vertical lines make the curve more pleasing to observe. Although the primary function of vertical lines is aesthetic, the distance between horizontal lines is crucial because they convey the change in cumulative probability. The following is an example of how the points of a survival curve could be roughly interpreted. Let us start to analyze the cumulative probability of surviving a given time could be read on the *Y*-axis. For example, the probability of surviving 28 months of the patients in the group labeled “T/T” is 60%; conversely, the probability of surviving the same time for patients belonging to the groups “C/T” and “C/C” is slightly more than 90%. It is worthy to note that the steepness of the curve is due to the absence of the event of interest (that is the length of horizontal lines). The censored patients are another element that impacts the survival point. Censored patients are indeed represented as tick marks on the survival curves. Censored values impact the cumulative probability of the groups under investigation. In details, the fourth and the fourteenth censored patients (represented by the ticks on the curve) into the “C/T” and “T/T” groups respectively, contribute in reducing the survival probability to live at least 28 months. Whereas, the fifth censored patients into the “C/C” group did not change the survival probability to live 28 months. However, the censored values contained in the three groups impact on reducing the cumulative survival among the intervals. Hence, we must be careful in interpreting anything beyond this point because our temporal data does not allow to extrapolate any further hypothesis on survival. It is worthy to note that intervals (horizontal lines) in the K and M curve are constructed only for the events of interest and not for the censored patients. As stated, this is conveyed in Figure 7 by means of the corner joining horizontal with vertical segments. Thus, in group “C/T”, “C/C”, and “T/T”, there are four, three, and nine events (vertical connections between the end of one interval and beginning of the next) demarcating five, four, and ten intervals (horizontals), respectively. It is worthy to note that there are no vertical changes due to the censored patients. Moreover, Figure 7 highlights in a remarkable way the capability of the K and M method to deal with variable intervals.

The comparison of survival curves is the most important step in all medical oncology clinical trial studies. The shape of the curve is important to evaluate. Curves that have many small steps usually have a higher number of participating subjects, whereas curves with large steps usually have a limited number of subjects and are, thus, less accurate. Whereas it is simple to visualize the difference between two survival curves, the difference must be quantified to assess statistical significance. The log-rank test and hazard ratio are the most common methods used for comparing survival curves. In detail, the log-rank test suggests whether two curves are statistically different, whereas the hazard ratio shows the increased rate of having an event in one curve versus the other.

## 4. Discussion

In this paper, we presented OSAnalyzer, a software tool to analyze correlations between patients’ outcome data and ADME gene variants as reported by the DMET microarray platform. Specifically, we developed a software able to compare simultaneously survival data according to each polymorphic variant of any of the 1936 SNPs evaluated by the DMET chip and rank the survival results according to the magnitude of the log-rank test significance. The latter is of particular interest due to the fact that this allows clinicians to focus their attention on the most important SNPs only. Furthermore, one of the main advantages of this software is that it is easy to include in prospective trial as a companion tool for the DMET platform, with the aim to associate clinical annotation with ADME gene SNPs. In the era of precision medicine this tool may represent a valuable resource to identify patients more likely to gain the best advantage from specific treatments in term of both PFS and OS.

## 5. Conclusions

We presented OSAnalyzer, an integrated software platform for the automatic generation and analysis of survival curves of patients subject to genotyping analysis using DMET microarray.

The automatic analysis of the whole microarray dataset avoids wasting time on the manual analysis of all probes in order to figure out which probes are relevant from an overall survival or PFS point of view.

OSAnalyzer, as opposed to SAM, survcomp, cBioPortal, Net-Cox, R, PSPP, and many other tools, makes it easy to analyze a whole DMET dataset, automatizing the pre-processing data steps. The currently available tools allow users to start the survival analysis only if the data are arranged in a compatible way with their data format. Thus, the pre-processing step could be error prone and time consuming because users have to manually arrange the data without being supported by the tool. Furthermore, all of the single setups have to be repeated for each row (gene) that has to be analyzed, forcing the users to manually investigate all the dataset to discover which rows are significant from a survival or PFS point of view. OSAnalyzer overcomes these issues providing to the user the *p*-value significance in term of PFS and overall survival in two different windows, sorted in descending order of *p*-value significance obtained by comparing the area under the curves by using the log-rank test.

## Figures and Tables

**Table 1:** A simple DMET (Drug Metabolizing Enzymes and Transporters) SNP (single nucleotide polymorphism) microarray dataset, where S and P, respectively, refer to the sample and probe identifiers.

	Samples	S_1_	S_2_	S_3_	...	S*_m_*
Probes						
P_1_		G/A	A/G	A/G	...	A/A
P_2_		T/C	C/C	T/T	...	T/C
⋮		⋮	⋮	⋮	⋮	⋮
P*_n_*		G/A	A/G	A/G	...	A/G

**Table 2:** A simple OS-dataset where, S and P respectively refer to sample and probe identifiers. OS refers to the collected OS time for each sample, Status-OS is a boolean variable where 1 means that the event was observed and 0 refers to censored data. PFS is a measure of the activity of a treatment on a disease. Status-PFS is a boolean variable where 1 means that the event was observed and 0 refers to censored data. PFS can only be measured in patients in which a tumor is present. Response is a boolean variable, where 1 means that the *i*-th sample presents metastasis and 0 refers to the absence of metastasis.

	Samples	S_1_	S_2_	S_3_	...	S*_m_*
Probes and OS Data						
OS		26.6	15.7	32.2	...	2.3
Status-OS		1	1	0	...	1
PFS		16.6	4.7	3.8	...	27.3
Status-PFS		1	0	0	...	1
Response		1	0	0	...	0
P_1_		G/A	A/G	A/G	...	A/A
P_2_		T/C	C/C	T/T	...	T/C
⋮		⋮	⋮	⋮	⋮	⋮
P*_n_*		G/A	A/G	A/G	...	A/A

## Abbreviations

The following abbreviations are used in this manuscript:

SNP	Single Polymorphism Nucleotide
K and M	Kaplan-Meier
DMET	(Drug Metabolizing Enzymes and Transporters)
ADME	(Absorption, Distribution, Metabolism, and Excretion)
NGS	Next Generation Sequencing
GWAS	Genome Wide-Association Studies
OS	Overall Survival Analysis
PFS	Progression-Free Survival
